# Assessment of the In Vivo Relationship Between Cerebral Hypometabolism, Tau Deposition, TSPO Expression, and Synaptic Density in a Tauopathy Mouse Model: a Multi-tracer PET Study

**DOI:** 10.1007/s12035-022-02793-8

**Published:** 2022-03-21

**Authors:** Heike Endepols, Marta Anglada-Huguet, Eckhard Mandelkow, Yannick Schmidt, Philipp Krapf, Boris D. Zlatopolskiy, Bernd Neumaier, Eva-Maria Mandelkow, Alexander Drzezga

**Affiliations:** 1 Institute of Radiochemistry and Experimental Molecular Imaging, Faculty of Medicine and University Hospital Cologne, University of Cologne, 50937 Cologne, Germany; 2 Institute of Neuroscience and Medicine, Nuclear Chemistry (INM-5), Forschungszentrum Jülich GmbH, Wilhelm-Johnen-Straße, 52428 Jülich, Germany; 3 Department of Nuclear Medicine, Faculty of Medicine and University Hospital Cologne, University of Cologne, 50937 Cologne, Germany; 4 CAESAR Research Center, DZNE (German Center for Neurodegenerative Diseases), Bonn, Germany; 5 Department of Neurodegenerative Diseases and Geriatric Psychiatry, University of Bonn, 53127 Bonn, Germany; 6 Max Planck Institute for Metabolism Research, 50931 Cologne, Germany; 7 Institute of Neuroscience and Medicine, Molecular Organization of the Brain (INM-2), Forschungszentrum Jülich GmbH, Wilhelm-Johnen-Straße, 52428 Jülich, Germany

**Keywords:** Small animal PET, Tau, Alzheimer’s disease, Neuroinflammation, Microglial activation, Synaptic density, Cerebral hypometabolism

## Abstract

Cerebral glucose hypometabolism is a typical hallmark of Alzheimer’s disease (AD), usually associated with ongoing neurodegeneration and neuronal dysfunction. However, underlying pathological processes are not fully understood and reproducibility in animal models is not well established. The aim of the present study was to investigate the regional interrelation of glucose hypometabolism measured by [^18^F]FDG positron emission tomography (PET) with various molecular targets of AD pathophysiology using the PET tracers [^18^F]PI-2620 for tau deposition, [^18^F]DPA-714 for TSPO expression associated with neuroinflammation, and [^18^F]UCB-H for synaptic density in a transgenic tauopathy mouse model. Seven-month-old rTg4510 mice (*n* = 8) and non-transgenic littermates (*n* = 8) were examined in a small animal PET scanner with the tracers listed above. Hypometabolism was observed throughout the forebrain of rTg4510 mice. Tau pathology, increased TSPO expression, and synaptic loss were co-localized in the cortex and hippocampus and correlated with hypometabolism. In the thalamus, however, hypometabolism occurred in the absence of tau-related pathology. Thus, cerebral hypometabolism was associated with two regionally distinct forms of molecular pathology: (1) characteristic neuropathology of the Alzheimer-type including synaptic degeneration and neuroinflammation co-localized with tau deposition in the cerebral cortex, and (2) pathological changes in the thalamus in the absence of other markers of AD pathophysiology, possibly reflecting downstream or remote adaptive processes which may affect functional connectivity. Our study demonstrates the feasibility of a multitracer approach to explore complex interactions of distinct AD-pathomechanisms in vivo in a small animal model. The observations demonstrate that multiple, spatially heterogeneous pathomechanisms can contribute to hypometabolism observed in AD mouse models and they motivate future longitudinal studies as well as the investigation of possibly comparable pathomechanisms in human patients.

**Supplementary Information:**

The online version contains supplementary material available at 10.1007/s12035-022-02793-8.

## Introduction

Alzheimer’s disease (AD) is characterized by the hallmarks of tau and amyloid-β deposits, associated with reactive gliosis, neuronal degeneration, neurotransmitter imbalance, and glucose hypometabolism [1, 2]. These pathologies can today be captured noninvasively in vivo, using molecular imaging techniques, namely positron emission tomography (PET) in combination with suitable radiotracers. PET tracers are applied in very low concentrations and therefore do not interfere with the physiological processes they visualize [3]. Employing several of these techniques in the same subjects in a multi-tracer approach allows to study interrelation of different pathomechanisms involved in AD, even in a longitudinal fashion. AD research already strongly relies on these advantages of in vivo molecular imaging in humans in order to study how AD pathomechanisms are intertwined with each other, as reflected in different cellular vulnerabilities, clinical phenotypes, and disease stages [4].

Of the mentioned methods, [^18^F]FDG-PET imaging represents the longest and best established technique to date. By measuring glucose metabolism, it is possible to estimate regional neuronal activity/dysfunction that is directly coupled to glucose metabolism/hypometabolism [5]. However, cerebral glucose metabolism and neuronal function are known to be influenced by many different factors. On the one hand, neuronal dysfunction may represent a common final pathway of neurodegeneration, potentially induced by different neurodegenerative processes such as co-localized protein aggregation pathologies, inflammation, and oxidative stress [6–8]. Loss of metabolic activity may be a result of synaptic dysfunction or density (in later stages also atrophy), but may also be due to regional hypoperfusion or direct metabolic factors such as insulin resistance [9]. Besides regional effects, hypometabolism/neuronal dysfunction may be caused remotely by means of impaired functional connectivity or diaschisis. To date, despite the availability of multimodal imaging tools, the exact pathomechanisms underlying hypometabolism observed in AD are not yet exhaustively investigated. This may in part also be due to limitations of multi-tracer protocols for human AD-patients, with regard to practicability, radiation exposure, and long follow-up intervals. Thus, animal studies represent a possibility to pursue these scientific questions.

However, available data on FDG-PET studies in small animal models of AD is partially inconsistent. In contrast to the consistent hypometabolism seen in human AD patients, both hypermetabolism and hypometabolism have been observed in transgenic AD mouse models [10]. This has frequently been attributed to increased glucose consumption of microglial cells due to neuroinflammation, possibly masking a concomitant hypometabolism in neurons [11]. In addition, certain intensity normalization methods, such as the use of the cerebellum as a reference region, may result in artificial findings of glucose hypermetabolism in AD mouse models [10] when the reference region itself was subject of metabolic changes.

The objective of the present multi-tracer PET study was to investigate the relationship between cerebral glucose metabolism and other neurodegenerative hallmark pathologies in a mouse model of Alzheimer’s disease. In detail, we aimed to ascertain the degree and regional extent of glucose utilization, and how this would be associated with tau deposition, increased TSPO expression (reflecting inflammation), and synaptic degeneration. For this project, we selected the transgenic tauopathy mouse model rTg4510. This well-characterized mouse line expresses human 0N4R-tau with the P301L mutation in the forebrain, leaving the cerebellum devoid of tau pathology [12].

Awake glucose metabolism was measured with [^18^F]fluorodeoxyglucose ([^18^F]FDG), which is widely used in the diagnosis of AD [8, 13, 14] and for studies in AD mouse models [10]. We selected an awake uptake protocol to avoid depression of neuronal activity by anesthesia [15].

In addition to measuring cerebral glucose metabolism, we decided to quantify regional tau burden. We preferred the assessment of tau over amyloid, as it has been previously demonstrated that tau aggregation pathology may have a more direct and regionally co-localized impact on neuronal function than amyloid-plaque aggregation [8]. Tau tangles were detected with the second generation tau tracer [^18^F]PI-2620. While other tau tracers have been used in rTg4510 mice [16, 17], [^18^F]PI-2620 has not yet been tested in AD mouse models. We selected this tracer because it has been reported to be superior to 1st-generation tau tracers in terms of high affinity for mixed 3R/4R but also for 4R isoforms of tau, and no off-target binding to monoamine oxidases (MAO-A/B) [18]. Neuroinflammation was measured with [^18^F]DPA-714, a second-generation ligand targeting the mitochondrial translocator protein 18 kDa (TSPO), which is expressed by activated microglia [19]. [^18^F]DPA-714 has been successfully employed in both AD patients [20, 21] and AD mouse models including rTg4510 mice [22–24]. Synaptic density was assessed with [^18^F]UCB-H which binds to the ubiquitous synaptic vesicle glycoprotein 2A (SV2A) [25, 26]. It has been used to measure synaptic density in AD patients [27] and rats [28], but so far not in AD mouse models. Previous studies have applied multimodal imaging approaches in small animal models, e.g., combining tau and inflammation PET imaging [16, 29]. However, to our knowledge, this is the first study combining all of the mentioned state-of-the-art tracers in a single animal model for imaging neurodegenerative pathologies in animals by means of a multi-tracer approach.

## Materials and Methods

### Animals

Experiments were carried out in accordance with the EU directive 2010/63/EU for animal experiments and the German Animal Welfare Act (TierSchG, 2006) and were approved by the regional authorities (LANUV NRW; application number 84–02.04.2015.A234 and 81–02.04.2018.A313).

The transgenic mouse model rTg4510 expresses the tau isoform 0N4R (Uniprot P-10636-D, alias “htau24”, 383 residues) with the FTDP17 mutation P301L [12, 30]. Briefly, rTg4510 animals were produced by crossing the activator mouse line CaMK2a-tTA (background C57BL/6), with the responder tetO.MAPT*P301L mouse line (background FVB). Mice having both the CaMK2a-tTA and tau transgene expressed human mutant P301L tau at high levels, up to ~ 13 times of murine tau. Mature tangles are present from 4 months of age [12]. Mice lacking the responder and the activator transgene were used as controls. Mice were screened by PCR using the following primer pairs: hTau transgene (Tau mPrP_E2): forward 5′-TGA ACC ATT TCA ACC GAG CTG-3′; reverse: 5′-TAC GTC CCA GCG TGA TCT TC-3′; CaMK2 promoter (oIMR8746/oIMR8747): forward 5′-CGC TGT GGG GCA TTT TAC TTT AG-3′; reverse: 5′-CAT GTC CAG ATC GAA ATC GTC-3′.

Sixteen adult mice were used for this study: eight rTg4510 mice (five females, three males; 22–29 g body weight) and eight non-transgenic littermates (four females, four males; 39–54 g body weight) at the age of 7 months. Brain weight is approx. 10% reduced at this age [12]. The mice were housed in groups in individually ventilated cages (NextGen, Ecoflow, Phantom, Allentown) under controlled ambient conditions (22 ± 1 °C and 55 ± 5% relative humidity) with a 12-h light/dark schedule and ad libitum access to food and water.

### PET Tracer Synthesis

[^18^F]FDG was purchased from Life Radiopharma Bonn GmbH. All other tracers were produced in-house. [^18^F]PI-2620 was synthesized according to Kroth et al. [18]. [^18^F]UCB-H was synthesized on a Trasis All-In-One synthesizer according to Warnier et al. [31].

For [^18^F]DPA-714, a novel procedure was developed: [^18^F]Fluoride was passed through an anion-exchange column (WatersSep-Pak Accell Light QMA cartridge). After fixation, [^18^F]fluoride was eluted and transferred to the reaction vessel with a solution of K_2_CO_3_ (1.8 mg in 200 µL water) and Kryptofix-222 (20 mg in 700 µL MeCN). The reaction mixture was evaporated to dryness at 100 °C within 15 min. After cooling to 50 °C *N,N*-diethyl-2-{2-[4-(2-toluenesulfonyloxyethoxy)phenyl]-5,7-dimethylpyrazolo[1,5-a]pyrimidin-3-yl}acetamide (4 mg dissolved in 700 µL DMSO) was added and allowed to react at 165 °C for 5 min. On completion, the crude product was diluted with sterile water and trapped on a Strata™-X-RP cartridge (30 mg/mL, Phenomenex), eluted with 1 mL MeCN and purified by HPLC. Data from 10 radiosyntheses showed that radiochemical yield (ndc) was 30.8 ± 4.4%, specific activity was 239 ± 30 GBq/µmol, and tracer mass was approx. 20 ng per injection. The identity of the ^18^F-labeled products was confirmed by HPLC with co-injection of the respective non-radioactive reference compound. (For spectral characterization of DPA-714 and the DPA-714 precursor, please see supplemental information.)

### Positron Emission Tomography (PET)

Although we aimed to examine each of the animals with all four tracers, this was not always possible due to technical reasons. Resulting sample sizes ranged from five to eight animals per group and tracer (see supplemental Tab. S1 for details).

The measurement procedures for [^18^F]PI-2620, [^18^F]DPA-714, and [^18^F]UCB-H were as follows: animals were anesthetized with isoflurane in O_2_/air 3:7 (induction 5%, maintenance 1.5–2.0%), and a catheter for tracer injection was inserted into the lateral tail vein. To this end, the tail was slightly warmed and a torniquet was applied with low pressure to the base of the tail. The catheters were home-made from a 30G tip broken off from a regular 30G needle and inserted into a flexible polyethylene tube with 0.28 mm inner diameter (Smiths Medical, Minneapolis, USA). After placement in the tail vein, the needle tip was secured with adhesive tape and the syringe with the tracer solution was attached to the other end of the tube. Mice were placed on an animal holder (medres® GmbH, Cologne, Germany) and fixed with a tooth bar in a respiratory mask. Body temperature was maintained at 37 °C using a feedback-controlled warming system. Eyes were protected from drying out by application of eye and nose ointment (Bepanthen, Bayer). A PET scan in list mode was conducted using a Focus 220 micro PET scanner (CTI-Siemens, Erlangen, Germany) with a resolution at the center of field of view of 1.4 mm. Data acquisition started with intravenous tracer injection (activity: 9–13 MBq in 125 µL) and lasted for either 30 min ([^18^F]DPA-714) or 60 min ([^18^F]PI-2620 and [^18^F]UCB-H). This was followed by a 10-min transmission scan using a ^57^Co point source for attenuation correction. After the scan was finished, the catheter was removed and the mice were returned to their home cage.

For [^18^F]FDG-PET, animals were briefly anesthetized (see above) and 9–12 MBq [^18^F]FDG in 125 µL was injected intraperitoneally. The mice were then placed in a solitary cage where they spent the following 35 min awake. Subsequently, they were anesthetized again and scanned as described above for 30 min. This protocol takes advantage of metabolic trapping of [^18^F]FDG [32], which allows awake tracer uptake and subsequent scanning under anesthesia [33, 34].

As we allowed for at least 5 days of recovery between the PET sessions, the mice tolerated the repeated measurements very well. During scanning, isoflurane anesthesia was as light as possible, with breathing rates of 60–80 per minute. During the waking phase (usually 1–2 min), the mice were kept on a warm plate and were closely monitored to reduce the risk of breathing arrest. The procedure for catheter placement described above caused only minimal scarring of the tail veins, so that multiple catheterizations were possible.

### Image Reconstruction

After full 3D rebinning, summed images were reconstructed using an iterative OSEM3D/MAP procedure [35], resulting in voxel sizes of 0.47 × 0.47 × 0.80 mm. Only in case of [^18^F]PI-2620, two frames of 30 min were reconstructed, and analysis was performed with the second frame. For all further processing of the images including statistics, the software VINCI 4.72 for MacOS X (Max Planck Institute for Metabolism Research, Cologne, Germany) was used. Images were co-registered and intensity-normalized to background. To this end, an elliptical volume of interest (VOI) of 4 mm^3^ was placed inside a background region (supplemental Fig. S1). For [^18^F]FDG and [^18^F]UCB-H, the cerebellum was chosen as reference region, since the tau transgene is not expressed in the cerebellum. Because of considerable spillover from the TSPO-expressing choroid plexus of the fourth ventricle, the cerebellum was not suitable as reference region for [^18^F]DPA-714. We therefore chose the midbrain, which was free of any spillover, as reference region for both [^18^F]DPA-714 and [^18^F]PI-2620 [36, 37]. Each image was divided by the mean value of the background VOI (supplemental Tab. S2), resulting in the “background-standardized uptake value ratio” (SUVR_bg_). No further postprocessing (e.g., Gauss filtering or spatial morphing) was done.

### Image Statistics

For comparison of rTg4510 versus controls, a voxel-wise *t*-test was performed for each tracer using VINCI 4.72 for MacOS X. The equation for the unpaired *t*-test (i.e., difference between the two group means divided by the pooled standard error of both groups) was executed manually in VINCI. The resulting t-maps were corrected for multiple comparisons using a threshold-free cluster enhancement (TFCE) procedure described in detail in the work of Smith and Nichols (2009) [38]. The TFCE procedure was implemented as a Python script in VINCI. For final thresholding, a permutation test with 10,000 permutations was performed in RStudio 1.0 for MaxOS X using the SUVR_bg_ values of the voxel with the highest TFCE value. The 95% quantile was calculated, and the corresponding TFCE level was used as the lower threshold of the t_TFCE_-map. The resulting t_TFCE_-maps were displayed in voxel view in shades of red (rTg4510 > controls) or blue (rTg4510 < controls) and projected onto an C57BL/6 T2-weighted MRI template. In addition, whole brain values were extracted from a whole brain VOI, which were used for *t*-tests in Graphpad Prism 6.0 for MacOS X. To assess the relationship between [^18^F]FDG or [^18^F]PI-2620 and the other tracers, Pearson correlation tests were performed at the voxel level across all animals (rTg4510 and controls pooled). This was followed by TFCE and permutation testing. Significance level was always *p* < 0.05.

### Autoradiography/Immunohistochemistry of Tau PHFs

To validate that [^18^F]Pi-2620 uptake reflects paired helical filaments (PHFs), we performed a combination of PET, autoradiography, and histology in one of the rTg4510 mice at the age of 11 months. 10.8 MBq [^18^F]PI-2620 was injected i.v. and a PET scan was performed for 30 min. The mouse was subsequently sacrificed; the brain was extracted from the scull and cut into two halves along the midline. One half was freshly frozen and cut into 50-µm sagittal sections on a cryostat. Sections were placed on a microscope slide, covered with scintillator foil and measured with a dFINE Betaimager (Biospace Lab, Paris, France) for 45 min. The measurement started 2 h and 15 min after [^18^F]PI-2620 injection.

The other half of the brain was immersed in 4% paraformaldehyde in PBS for 72 h and then transferred to a 30% sucrose solution. Subsequently, it was processed for thioflavin S (ThioS) staining and PFH1 immunohistochemistry. For ThioS staining, 35-µm sagittal brain sections were washed in 70% and 80% EtOH and incubated with ThioS solution (1% in 80% EtOH) for 15 min. Sections were washed with 80% EtOH, 70% EtOH, distilled water and mounted in aqueous mounting media.

PHF1 immunohistochemistry with diaminobenzidine (DAB) as chromogen was performed as follows: endogenous peroxidases were blocked in PBS containing 1% methanol and 0.6% H_2_O_2_. Then, nonspecific protein interactions were blocked with normal serum (4%) in PBS with 0.1% Triton x-100. PHF1 antibody (1:60; gifts from Dr. P. Davies, Albert Einstein College, Bronx, NY) was incubated overnight at 4 °C. For the secondary antibody and avidin-biotinylated peroxidase system, we used the Vectastain Universal Elite ABC kit (Vector Laboratories) and for developing the DAB Quanto Chromogen (ThermoFisher).

## Results

### Autoradiography/Immunohistochemistry of Tau PHFs

**Fig. 1:**
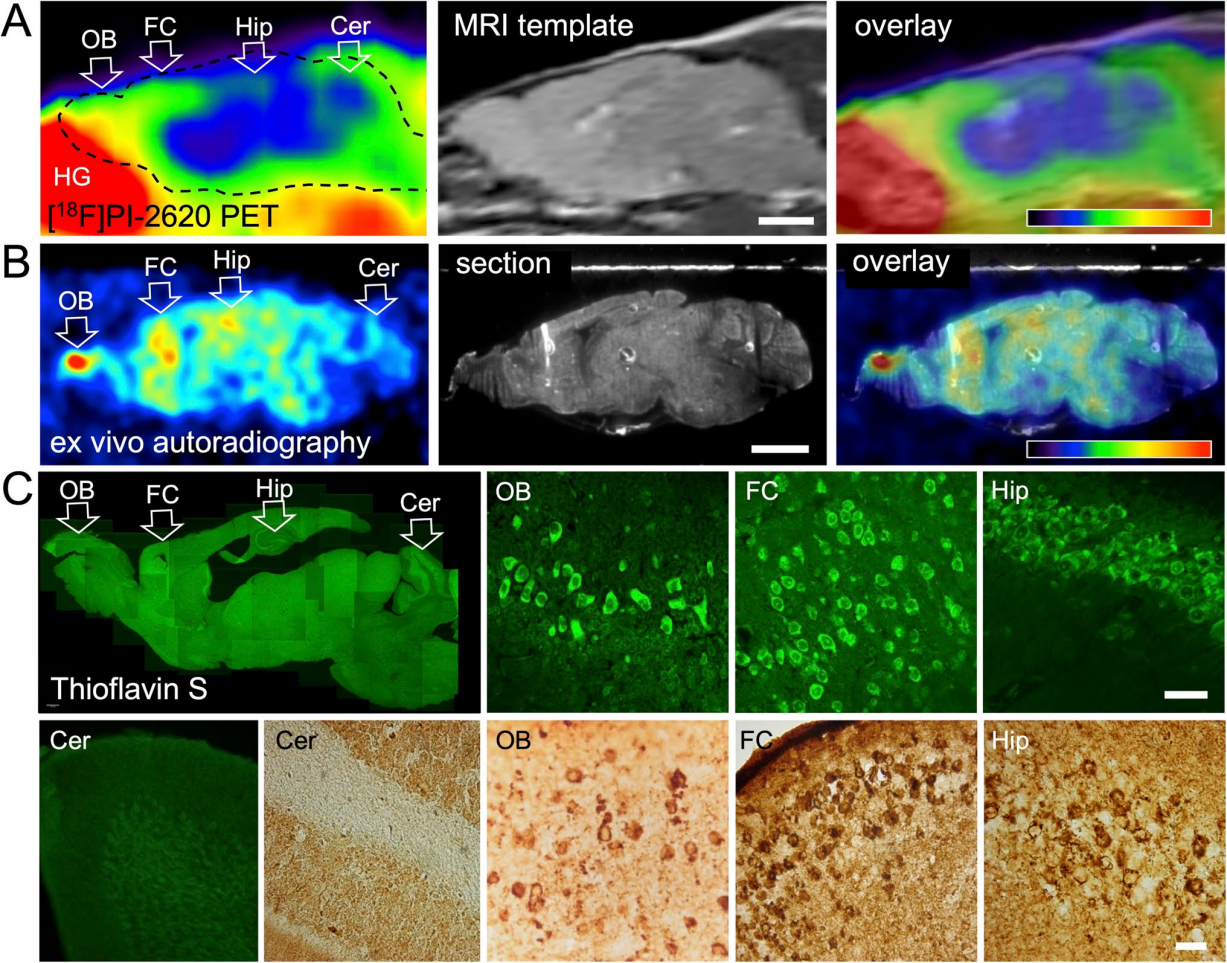
Comparison of [^18^F]PI-2620 PET, ex vivo autoradiography and histology. Shown are sagittal sections of the same mouse (11 months old), rostral = left. (A) [^18^F]PI-2620 PET, start 8 min after tracer injection, 30-min scan duration. Scale bar: 2.5 mm. Color scale: 0–5 SUVR_bg_. Tracer accumulation was highest in the olfactory bulb and the frontal cortex. (B) Ex vivo autoradiography of the same animal, start 2 h 15 min after injection, duration 45 min. Smoothing 0.61 mm FWHM. Scale bar: 2.5 mm. Color scale: 0.000–0.0015 cpm. Highest tracer accumulation is visible in the olfactory bulb, frontal cortex, and hippocampus. (C) Histology of the same animal: Thioflavin S staining (green) and PHF1 staining (brown). Scale bars: 30 µm. Tau fibrils are present in the olfactory bulb, frontal cortex, and hippocampus, but absent in the cerebellum. Abbreviations: Cer, cerebellum; FC, frontal cortex; HG, Harderian gland; Hip, hippocampus; OB, olfactory bulb

[^18^F]PI-2620 PET showed high uptake in the olfactory bulb and frontal cortex, and medium uptake in the hippocampus (Fig. 1A). This was confirmed by autoradiography (Fig. 1B). The Harderian glands, which accumulate [^18^F]PI-2620 strongly, had been removed before autoradiography. The spot with high [^18^F]PI-2620 uptake in the olfactory bulb became even more noticeable without the Harderian gland spillover. Both ThioS and the PHF1 antibody revealed numerous densely stained cell bodies in the olfactory bulb, indicating the presence of tau PHFs (Fig. 1C). A high density of neurons with PHF deposits were also found in the frontal cortex and the hippocampus, while the cerebellum was largely PHF-negative (Fig. 1C).

### Cerebral Glucose Metabolism Measured with [^18^F]FDG (Fig. 2A)

**Fig. 2:**
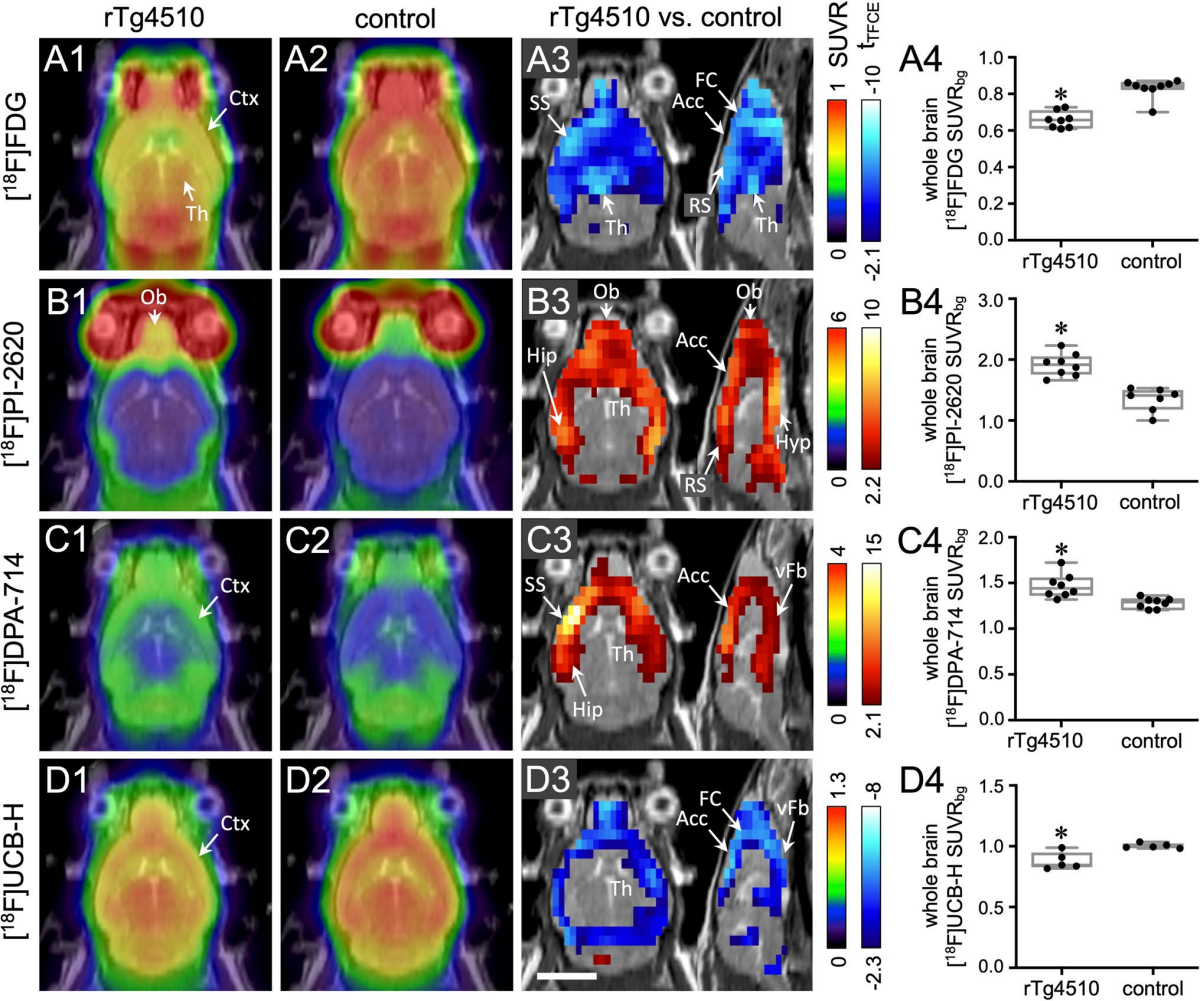
Multitracer imaging of rTg4510 mice and non-transgenic littermates (controls). Each row A–D represents a different tracer. Column 1: mean horizontal images of rTg4510 mice (*n* = 5 to 8). Column 2: mean images of controls (*n* = 5 to 8). Column 3: voxel-wise comparison between rTg4510 and control mice (*t*-test, corrected for multiple testing). Red and blue voxels indicate significantly (*p* < 0.05) higher and lower tracer uptake, respectively, in rTg4510 mice. Column 4: comparison of whole brain tracer uptake (*t*-test). *t-test, *p* < 0.05. Scale bar: 5 mm. Abbreviations: Acc, anterior cingulate cortex; Ctx, cortex; FC, frontal cortex; Hip, hippocampus; Hyp, hypothalamus; Ob, olfactory bulb; RS, retrosplenial cortex; SS, somatosensory cortex; Th, thalamus; vFb, ventral forebrain

[^18^F]FDG uptake was significantly lower in the whole forebrain of rTg4510 mice (Fig. 2A3). Areas with the highest decrease were the frontal cortex, cingulate cortex, amygdala, and thalamus. Whole brain uptake of [^18^F]FDG was also significantly lower in rTg4510 mice (SUVR_bg_ 0.66 ± 0.04) compared to controls (SUVR_bg_ 0.83 ± 0.05; *p* < 0.0001; Fig. 1A4).

### Tau Accumulation Measured with [^18^F]PI-2620 (Fig. 2B)

Uptake of [^18^F]PI-2620 was significantly higher in rTg4510 mice compared to controls in all cortical regions, hippocampus and the ventral forebrain, but not in the thalamus (Fig. 2B3). Overall, significant differences were more prominent in posterior cortical areas, i.e., entorhinal and retrosplenial cortex. A high uptake of [^18^F]PI-2620 was observed in the olfactory bulb in all rTg4510 mice (Fig. 2B1) whereas only two control mice showed high accumulation in this region. Whole brain uptake of [^18^F]PI-2620 was significantly higher in rTg4510 mice (mean SUVR_bg_ ± standard deviation: 1.91 ± 0.19) compared to controls (SUVR_bg_ 1.34 ± 0.19; *p* < 0.05; Fig. 2B4). The tracer distribution was consistent with the pattern of tau accumulation observed in ex vivo autoradiography, PHF immunohistochemistry, and ThioS staining experiments (Fig. 1).

### TSPO Expression Measured with [^18^F]DPA-714 (Fig. 2C)

A significantly higher uptake of [^18^F]DPA-714 in all cortical regions, hippocampus and the ventral forebrain was observed in rTg4510 mice. The highest tracer accumulation was found in the frontal cortex and anterior somatosensory cortex (Fig. 2C3). The dorsal thalamus and brainstem did not accumulate [^18^F]DPA-714. Whole brain uptake of [^18^F]DPA-714 was significantly higher in rTg4510 mice (SUVR_bg_ 1.53 ± 0.16) compared to controls (SUVR_bg_ 1.35 ± 0.07; *p* < 0.05; Fig. 2C4).

### Synaptic Density Measured with [^18^F]UCB-H (Fig. 2D)

In rTg4510 mice, a significantly lower uptake of [^18^F]UCB-H was observed in the frontal, medial, and parietal cortex and the ventral forebrain, but not in the hippocampus, thalamus, cerebellum, and brainstem (Fig. 2D3). Whole brain uptake of [^18^F]UCB-H was also significantly lower in rTg4510 mice (SUVR_bg_ 0.88 ± 0.07) when compared to controls (SUVR_bg_ 1.00 ± 0.02; *p* < 0.05; Fig. 2D4).

### Correlation Analyses

**Fig. 3:**
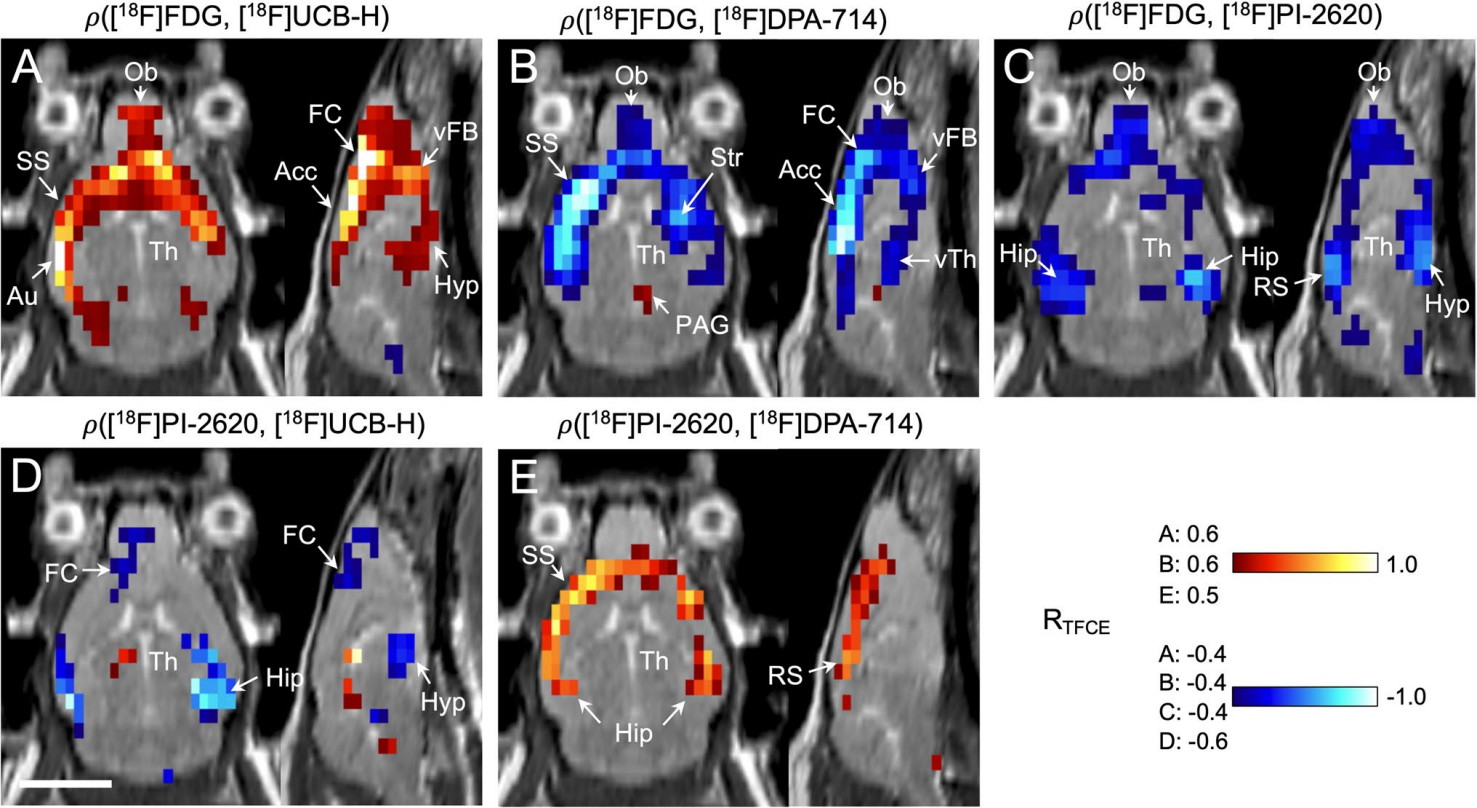
Correlation between uptake of different tracers in rTg4510 mice and controls (pooled). Datasets of different tracers were correlated with each other using a Pearson correlation test (significance level *p* < 0.05, corrected for multiple testing). The resulting correlation maps show for each voxel if the two respective tracers are positively (red) or negatively correlated (blue), or show no relationship at all (gray). The strength of correlation is coded by hue, with light colors reporting strong correlation (R near ± 1) and dark colors indicating weak correlation (R near the significance threshold). The respective significance thresholds are noted on the left side of the color bars, and the brain areas with significant clusters are labeled. [^18^F]FDG (glucose metabolism) was correlated with (A) [^18^F]UCB-H (synaptic density), (B) [^18^F]DPA-714 (TSPO expression), and (C) [^18^F]PI-2620 (tau deposition). [^18^F]PI-2620 (tau deposition) was correlated with (D) [^18^F]UCB-H (synaptic density) and (E) [^18^F]DPA-714 (TSPO expression). Scale bar: 5 mm. Abbreviations: Acc, anterior cingulate cortex; Au, auditory cortex; FC, frontal cortex; Hip, hippocampus; Hyp, hypothalamus; Ob, olfactory bulb; PAG, periaqueductal gray; R_TFCE_, correlation coefficient; thresholded with a TFCE procedure [38]; RS, retrosplenial cortex; SS, somatosensory cortex; Str, striatum; Th, thalamus; vFb, ventral forebrain; vTh, ventral thalamus

The relationship between the imaging biomarkers in rTg4510 mice was further examined by correlation analysis. [^18^F]FDG and [^18^F]UCB-H uptake was mostly positively related (Fig. 3A), comprising somatosensory, auditory, frontal, and anterior cingulate cortex, basal forebrain, and hypothalamus. [^18^F]FDG and [^18^F]UCB-H uptake was correlated in the thalamus midline only, while all other thalamic nuclei showed no relationship between glucose metabolism and synaptic density.

The correlation pattern between [^18^F]FDG and [^18^F]DPA-714 uptake looked almost like a mirror image of the correlation between [^18^F]FDG and [^18^F]UCB-H uptake. Significant negative correlations were detected in the frontal, somatosensory, and anterior cingulate cortex, as well as in the olfactory bulb and basal forebrain (Fig. 3B). A small cluster of positive correlation emerged in the periaqueductal gray, which disappeared after atrophy correction (Supplemental Fig. 7).

The relationship between [^18^F]FDG and [^18^F]PI-2620 uptake was not as pronounced as that of the aforementioned tracers (Fig. 3C). A negative correlation was observed in the hippocampus, amygdala, hypothalamus, olfactory bulb, and retrosplenial cortex. The other cortical areas showed weak or no correlation.

Correlation between [^18^F]PI-2620 and [^18^F]UCB-H uptake (Fig. 3D) looked similar to the correlation pattern of [^18^F]FDG and [^18^F]PI-2620 uptake. Significant negative relationships occurred in the hippocampus, amygdala, hypothalamus, and frontal and somatosensory cortex.

The correlation between [^18^F]PI-2620 and [^18^F]DPA-714 was confined to the cortex and hippocampus (Fig. 3E).

## Discussion

For the first time, in vivo imaging of tau aggregates has been successfully performed using the tracer [^18^F]PI-2620 in the rTg4510 mouse model, confirmed by ex vivo autoradiography and immunohistochemistry. Likewise, the synaptic tracer [^18^F]UCB-H was applied to this mouse model, and both tracers were combined with [^18^F]DPA-714 (TSPO expression) and [^18^F]FDG (glucose metabolism) [1]. The present multitracer PET study revealed (1) glucose hypometabolism in the brain, which is a common hallmark of human AD but not consistent in AD mouse models [10], can also be observed in the whole forebrain of rTg4510 mice. (2) Tau and (3) TSPO expression (i.e., neuroinflammation) was found in the olfactory bulb, cortex, hippocampus, and ventral forebrain. (4) Synaptic density was reduced in mostly the same regions. Based on the correlation analyses, cerebral hypometabolism observed in these mice may be attributable to two different underlying pathomechanisms, located in regionally distinct anatomical brain regions.

The first pathomechanism refers to a set of anatomical regions comprising the cortex, hippocampus, basal forebrain, and hypothalamus. In these regions predominantly a colocalization (and quantitative correlation) of pathologies typically associated with AD-neurodegeneration (tau aggregation, synaptic loss, and inflammation) and hypometabolism was observed. These results indicate that hypometabolism in these regions may indeed represent neuronal dysfunction linked to AD-type neuropathology. In particular, regions of reduced synaptic density also showed reduced [^18^F]FDG uptake. Because synaptic activity accounts for 70–80% of brain glucose consumption [39–42], [^18^F]FDG PET is very sensitive for synaptic degeneration [43]. Furthermore, in the same brain regions, a negative correlation between [^18^F]DPA-714 and [^18^F]FDG uptake was observed, which is in line with previous evidence that increased TSPO expression is associated with synaptic loss [44–46] and hence with reduced glucose metabolism.

[^18^F]PI-2620 uptake (indicating tau aggregation) was significantly increased in the same brain regions where neuroinflammation and synaptic loss took place. Ex vivo autoradiography with [^18^F]PI-2620, PHF immunohistochemistry, and ThioS staining suggested that mature neurofibrillary tangles were indeed present in the brain regions with increased [^18^F]PI-2620 uptake. A correlation between [^18^F]PI-2620 and [^18^F]DPA-714 was found in the cortex and hippocampus, which is consistent with earlier studies with the TSPO-tracer [^11^C]AC-5216 [29, 47] in rTg4510 mice. Notably, [^18^F]PI-2620 and [^18^F]DPA-714 uptake were not correlated in the ventral forebrain, olfactory bulb, and hypothalamus, although tau deposition and increased TSPO expression were both present in rTg4510 mice. This can be explained by the fact that the interaction between tau pathology and increased TSPO expression is complex; i.e., tau pathology can enhance or attenuate the neuroinflammatory response [48], and the two processes can follow different time courses [49]. Besides, there may be other factors, e.g., tau oligomers [50] or the local microglia density [51], that influence the magnitude of neuroinflammation.

Taken together, in large proportions of the brain of the transgenic animals, a typical pathomechanistic cascade was demonstrated in this multitracer setup, consistent with common hypotheses on the development of Alzheimer’ disease. In regional colocalization with increased tau aggregation in the cortex and hippocampus, increased TSPO expression as well as synaptic loss was observed as well as locally reduced glucose metabolism. These findings support the notion that hypometabolism in these regions may represent neuronal dysfunction in consequence to tau aggregation leading to inflammation and synaptic loss. Importantly, these findings also imply that the chosen experimental setup may be valuable to study the mentioned pathologies and their interactions in vivo in a small animal model also longitudinally.

A second type of pathological phenomenon comprised strong hypometabolism in the thalamus, which was not accompanied by reduced [^18^F]UCB-H uptake, and did therefore not indicate an alteration of synaptic density. Moreover, neither pathologically increased tau accumulation nor increased TSPO expression was found in the thalamus. Thus, the observed hypometabolism was independent of characteristic markers of neurodegeneration in this region. One possible explanation is that thalamic hypometabolism may represent a network-related phenomenon, i.e., an adaptive/compensatory downstream consequence of remote neurodegenerative processes resulting in reduced functional input to the thalamus. In contrast to frontotemporal lobar degeneration [52] and corticobasal degeneration [53], major thalamic hypometabolism is not a characteristic finding in AD; therefore, attention is usually not drawn to this region. Although it is not generally regarded as a representative feature of AD, there are nevertheless some studies demonstrating thalamic hypometabolism related to AD [54–56]. Furthermore, during the course of AD, considerable remodeling of functional connectivity takes place [57]. In patients suffering from mild cognitive impairment, decreased integrity of thalamus-related networks has been shown, including thalamo-hippocampal and thalamo-cortical connections [58, 59]. A general reduction of functional connectivity has also been found in AD mouse models [60]. A less active network consumes less energy, which would explain the reduced thalamic [^18^F]FDG uptake. However, to date we can only speculate about the nature of the underlying pathological process, and we cannot rule out that it is partly caused by disruption of the mouse Fgf14 gene in the rTg4510 model. We will therefore address this intriguing finding in future studies. Provided that the existence of two distinct pathological processes underlying the decreased glucose consumption in spatially segregated brain regions can be confirmed, this could shed new light on the complex pathophysiology of AD and even have certain therapeutic implications. Thus, if cerebral hypometabolism in the thalamus as observed in the present study is actually related to AD but not a consequence of local tau pathology and neurodegeneration, it might be reversible and thus amenable to therapy.

## Conclusion

The present study demonstrates that multi-tracer PET imaging can provide important complementary information about different pathomechanisms and their complex relationship in neurodegenerative disorders. We showed that 7-month-old rTg4510 mice consistently exhibit glucose hypometabolism in the entire forebrain. Interestingly, other neurodegenerative pathologies were not homogeneously associated with the detected hypometabolism; instead two spatially segregated phenomena were observed: (1) in neocortex and hippocampus of the transgenic animals, a typical neurodegenerative cascade was found, including tau deposition associated with neuroinflammation and reduced synaptic density in colocalization with local hypometabolism. These findings are consistent with common hypotheses on development of AD. (2) In the thalamus of the animal models, distinct hypometabolism in the absence of other signs of neurodegeneration was observed, possibly reflecting downstream/remote adaptive processes. Because these changes found in the thalamus were not directly related to standard hallmarks of neurodegeneration, they may be reversible and potentially of therapeutic value. Together these findings underscore the value of studying regional interaction and remote effects of AD-neuropathology in a small animal model in vivo, including potential longitudinal approaches. Furthermore, the results motivate efforts to replicate similar investigations in human patients.

## Supplementary Information

Below is the link to the electronic supplementary material.

Supplementary file1 (DOCX 2.73 MB)

## Data Availability

The datasets used and/or analyzed during the current study are available from the corresponding author on reasonable request.
