# Supplementary material for: Assessment of the In Vivo Relationship Between Cerebral Hypometabolism, Tau Deposition, TSPO Expression, and Synaptic Density in a Tauopathy Mouse Model: a Multi-tracer PET Study
Source: Mol Neurobiol. 2022 Mar 21;59(6):3402–13. doi: 10.1007/s12035-022-02793-8 (PMC9148291; doi:10.1007/s12035-022-02793-8)

**Supplemental information**

Title: Assessment of the *in vivo* relationship between cerebral hypometabolism and Tau-deposition, TSPO-expression, and synaptic density in a tauopathy mouse model: A multi-tracer PET study

Authors: Heike Endepols, Marta Anglada-Huguet, Eckhard Mandelkow, Yannick Schmidt, Philipp Krapf, Boris D. Zlatopolskiy, Bernd Neumaier, Eva-Maria Mandelkow, Alexander Drzezga

**Supplemental methods**

Sample sizes

Supplemental Table S1: Tracer targets and sample sizes

| Tracer | Target | rTg4510 | controls |
| --- | --- | --- | --- |
| [^18^F]PI-2620 | aggregated Tau | n=8 | n=7 |
| [^18^F]DPA-714 | TSPO 18 kDa | n=8 | n=8 |
| [^18^F]UCB-H | SV2A | n=5 | n=5 |
| [^18^F]FDG | glucose metabolism | n=8 | n=8 |

VOIs used for intensity normalization

For intensity normalization, different reference areas were selected for the different tracers.

This was the cerebellum for [^18^F]FDG and [^18^F]UCB-H, midbrain for [^18^F]PI-2620 and [^18^F]DPA-714. An elliptical VOI (4 mm^3^) was placed in the respective reference region (Suppl. Fig. S1), and the activity in Bq/ccm^3^ was extracted (Suppl. Tab. 2). Each image was then divided by its reference value, yielding the dimensionless SUVR.


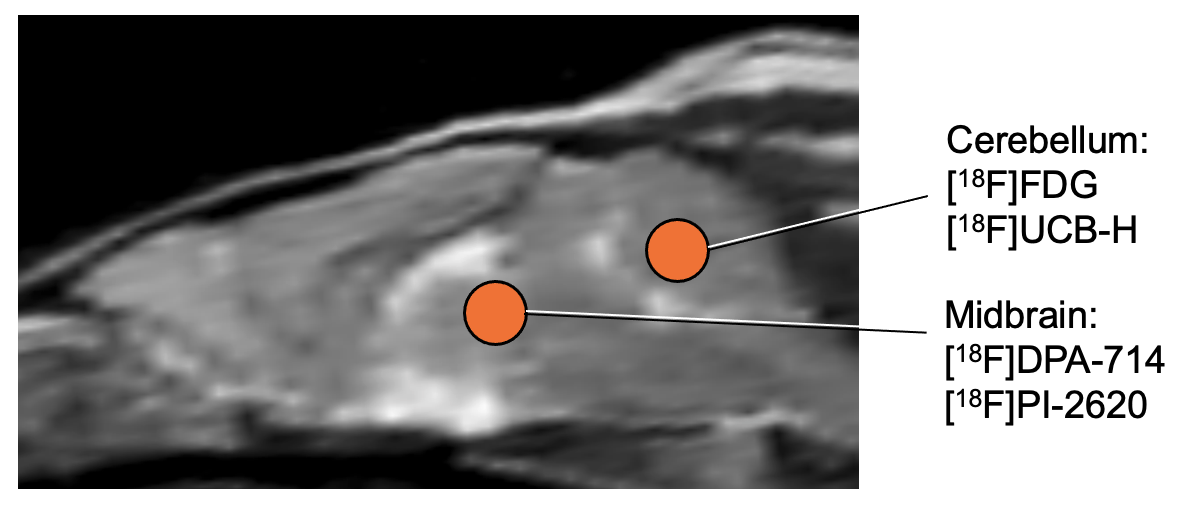


Supplemental Fig. S1: VOIs used for intensity normalization.

Supplemental Table S2: VOI values in Bq/cm^3^ for intensity normalization

| animal # | [^18^F]FDG, cerebellum VOI | [^18^F]PI-2620,  midbrain VOI | [^18^F]DPA-714,  midbrain VOI | [^18^F]UCB-H,  olfactory VOI |
| --- | --- | --- | --- | --- |
| Tg0987 | 284054 | 17575 | 193740 | n.m. |
| Tg0988 | 265603 | 17321 | 136147 | n.m. |
| Tg1081 | 305634 | 17671 | 58897 | 254637 |
| Tg1083 | 206211 | 12777 | 57093 | 213701 |
| Tg1088 | 219207 | 19470 | 66104 | n.m. |
| Tg1251 | 171267 | 23237 | 176389 | 344913 |
| Tg1254 | 298855 | 17398 | 105260 | 287350 |
| Tg1256 | ﻿216574 | 20195 | 111072 | 356233 |
| Ct0985 | 214228 | 16229 | 96362 | n.m. |
| Ct0945 | 245007 | 13633 | 127863 | n.m. |
| Ct1082 | 173272 | 11159 | 62902 | 232598 |
| Ct1085 | 162486 | 14553 | 57423 | 297481 |
| Ct1091 | 298557 | n.m. | 87589 | n.m. |
| Ct1248 | 178977 | 15440 | 97345 | 252652 |
| Ct1252 | 117087 | 21196 | 88046 | 182190 |
| Ct1255 | 239080 | 15258 | 100637 | 260594 |

n.m. not measured

**Supplemental results**

Atrophy correction

1.) VOI-based analysis using VOIs reduced in size.

A VOI-based comparison of tracer uptake between rTg4510 mice and controls was performed with full-sized VOIs and with VOIs reduced in size to account for brain atrophy. A two-way ANOVA was used with factors "transgene" (factor levels: rTg4510 and controls) and "brain area" (repeated measures factor with factor levels: thalamus, hypothalamus, hippocampus, retrosplenial cortex, anterior cingulate cortex, frontal cortex, somatosensory cortex). VOIs are shown in Suppl Fig. S2 and S3, and statistical values can be found in supplemental tables S3 – S6. Results were the same for both approaches, except for the comparison of [^18^F]DPA-714 uptake in the retrosplenial cortex, which was significant for the small VOIs, but not for the full-sized VOIs.

Supplemental Table S3: VOI-based results for [^18^F]FDG, n=8 rTg4510 + 8 controls

| [^18^F]FDG | full-size VOIs | small VOIs |
| --- | --- | --- |
| main effect transgene | F(1,14)=43.53, p<0.0001 | F(1,14)=44.29, p<0.0001 |
| main effect brain area | F(6,84)=63.87, p<0.0001 | F(6,84)=73.82, p<0.0001 |
| factor interaction | F(6,84)=14.50, p<0.0001 | F(6,84)=15.84, p<0.0001 |
| **rTg4510 vs. controls** |  |  |
| Thalamus | **p=0.0037** | **p=0.0071** |
| Hypothalamus | p=0.0661 | p=0.1389 |
| Hippocampus | **p=0.0007** | **p=0.0023** |
| Retrosplenial cortex | **p<0.0001** | **p<0.0001** |
| Anterior cingulate cortex | **p<0.0001** | **p<0.0001** |
| Frontal cortex | **p<0.0001** | **p<0.0001** |
| Somatosensory cortex | **p<0.0001** | **p<0.0001** |

Supplemental Table S4: VOI-based results for [^18^F]PI-2620, n=8 rTg4510 + 7 controls

| [^18^F]PI-2620 | full-size VOIs | small VOIs |
| --- | --- | --- |
| main effect transgene | F(1,13)=40.67, p<0.0001 | F(1,13)=36.39, p<0.0001 |
| main effect brain area | F(6,78)=46.87, p<0.0001 | F(6,78)=36.73, p<0.0001 |
| factor interaction | F(6,78)=10.18, p<0.0001 | F(6,78)=7.96, p<0.0001 |
| **rTg4510 vs. controls** |  |  |
| Thalamus | p>0.9999 | p=0.9970 |
| Hypothalamus | **p<0.0001** | **p=0.0004** |
| Hippocampus | p=0.1072 | p=0.3388 |
| Retrosplenial cortex | p=0.1900 | p=0.1872 |
| Anterior cingulate cortex | **p=0.0004** | **p=0.0034** |
| Frontal cortex | **p<0.0001** | **p<0.0001** |
| Somatosensory cortex | p=0.0589 | p=0.2367 |

Supplemental Table S5: VOI-based results for [^18^F]DPA-714, n=8 rTg4510 + 8 controls

| [^18^F]DPA-714 | full-size VOIs | small VOIs |
| --- | --- | --- |
| main effect transgene | F(1,14)=31.14, p<0.0001 | F(1,14)=29.85, p<0.0001 |
| main effect brain area | F(6,84)=322.0, p<0.0001 | F(6,84)=312.5, p<0.0001 |
| factor interaction | F(6,84)=9.85, p<0.0001 | F(6,84)=9.34, p<0.0001 |
| **rTg4510 vs. controls** |  |  |
| Thalamus | p=0.2591 | p=0.9923 |
| Hypothalamus | **p<0.0001** | **p=0.0288** |
| Hippocampus | **p=0.0100** | **p=0.0107** |
| Retrosplenial cortex | p=0.2727 | **p=0.0137 #** |
| Anterior cingulate cortex | **p<0.0001** | **p<0.0001** |
| Frontal cortex | **p<0.0001** | **p<0.0001** |
| Somatosensory cortex | **p<0.0001** | **p<0.0001** |

#: Difference between full-sized and small VOIs

Supplemental Table S6: VOI-based results for [^18^F]UCB-H, n=5 rTg4510 + 5 controls

| [^18^F]UCB-H | full-size VOIs | small VOIs |
| --- | --- | --- |
| main effect transgene | F(1,8)=18.47, p=0.0026 | F(1,8)=17.10, p=0.0033 |
| main effect brain area | F(6,48)=33.50, p<0.0001 | F(6,48)=39.41, p<0.0001 |
| factor interaction | F(6,48)=4.83, p=0.0006 | F(6,48)=5.04, p=0.0004 |
| **rTg4510 vs. controls** |  |  |
| Thalamus | p=0.9651 | p=0.9993 |
| Hypothalamus | **p=0.0006** | **p=0.0001** |
| Hippocampus | p=0.5276 | p=0.6204 |
| Retrosplenial cortex | **p=0.0064** | **p=0.0444** |
| Anterior cingulate cortex | **p<0.0001** | **p<0.0001** |
| Frontal cortex | **p=0.0008** | **p=0.0046** |
| Somatosensory cortex | **p=0.0037** | **p=0.0041** |


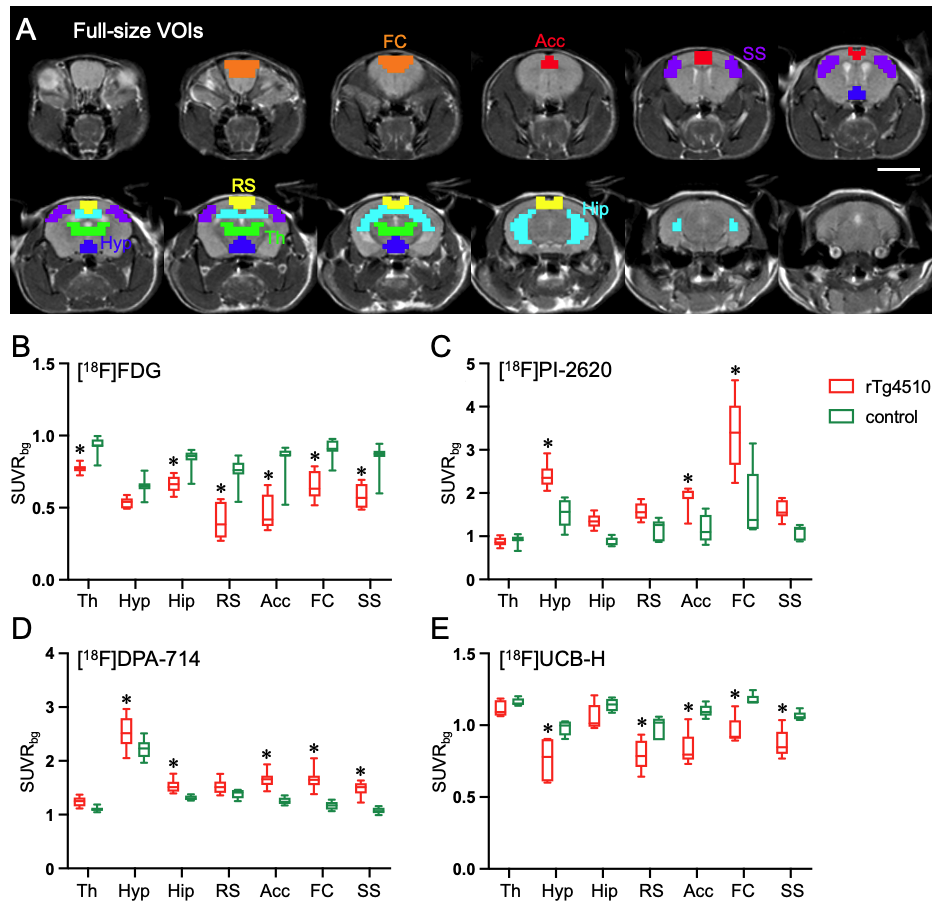


Supplemental Fig. S2: VOI-based analysis with full-sized VOIs using the original images (no resizing of the rTg4510 brains).

A: Localization of VOIs

B: Regional comparison of [^18^F]FDG uptake between rTg4510 mice and controls (two-way ANOVA).

C: Regional comparison of [^18^F]PI-2620 uptake between rTg4510 mice and controls (two-way ANOVA).

D: Regional comparison of [^18^F]DPA-714 uptake between rTg4510 mice and controls (two-way ANOVA).

E: Regional comparison of [^18^F]UCB-H uptake between rTg4510 mice and controls (two-way ANOVA).

* p<0.05 for post-hoc testing (Sidak's).

Abbreviations: Acc: anterior cingulate cortex, FC: frontal cortex, Hip: hippocampus, Hyp: hypothalamus, RS: retrosplenial cortex, SS: somatosensory cortex; Th: thalamus. Scale bar: 5 mm.


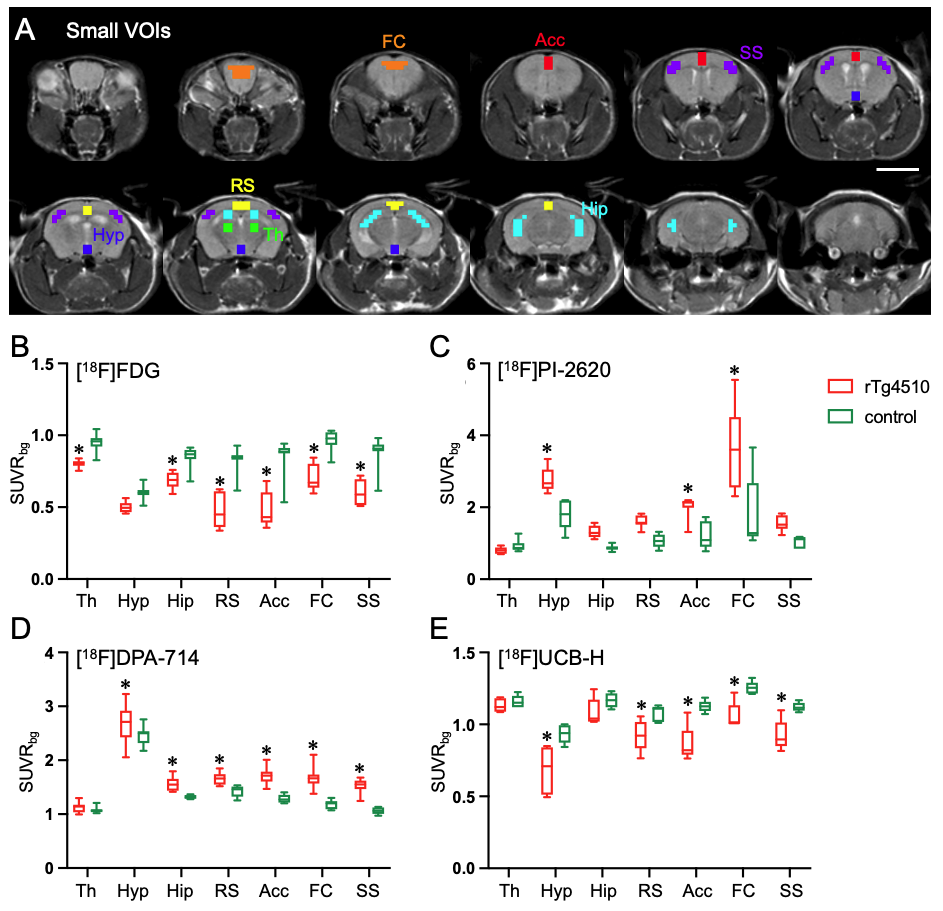


Supplemental Fig. S3: VOI-based analysis with VOIs of reduced size, using the original images (no resizing of the rTg4510 brains).

A: Localization of VOIs

B: Regional comparison of [^18^F]FDG uptake between rTg4510 mice and controls (two-way ANOVA).

C: Regional comparison of [^18^F]PI-2620 uptake between rTg4510 mice and controls (two-way ANOVA).

D: Regional comparison of [^18^F]DPA-714 uptake between rTg4510 mice and controls (two-way ANOVA).

E: Regional comparison of [^18^F]UCB-H uptake between rTg4510 mice and controls (two-way ANOVA).

* p<0.05 for post-hoc testing (Sidak's).

Abbreviations: Acc: anterior cingulate cortex, FC: frontal cortex, Hip: hippocampus, Hyp: hypothalamus, RS: retrosplenial cortex, SS: somatosensory cortex; Th: thalamus. Scale bar: 5 mm.

With respect to correlation between the uptake of the different tracers, thalamus and frontal cortex were selected as examples of the two emerging correlation patterns. The thalamus showed a significant hypometabolism, but neither significant tau deposition, neuroinflammation nor synaptic loss. Consequently, thalamic [^18^F]FDG uptake was not correlated to [^18^F]UCB-H and [^18^F]DPA-714 uptake, and also no correlation of [^18^F]PI-2620 and [^18^F]DPA-714 uptake was found, neither with full-sized VOIs nor with VOIs of reduced size (Supplemental table S7, Supplemental figures S5 and S6). The only significant correlation [^18^F]FDG vs. [^18^F]PI-2620 with the full-sized VOI disappeared when a thalamic VOI of reduced size was used.

The frontal cortex suffered from significant hypometabolism as well, and also showed significant tau deposition, inflammation and synaptic loss. [^18^F]FDG uptake was correlated to all other tracers, regardless of VOI size. [^18^F]PI-2620 uptake was correlated to [^18^F]UCB-H and [^18^F]DPA-714 uptake with the full-sized PFC VOI and to [^18^F]DPA-714 uptake with the small VOI (Supplemental table S7, Supplemental figures S4 and S5).

Supplemental table S7: Correlation of tracer uptake in the thalamus and frontal cortex (FC).

| Correlation | [^18^F]FDG vs. [^18^F]UCB-H | [^18^F]FDG vs. [^18^F]DPA-714 | [^18^F]FDG vs. [^18^F]PI-2620 | [^18^F]PI-2620 vs. [^18^F]UCB-H | [^18^F]PI-2620 vs. [^18^F]DPA-714 |
| --- | --- | --- | --- | --- | --- |
| Thalamus  full-size VOI | R=0.47  p=0.1744 | **R= -0.62**  **p=0.0098** | R= 0.12  p=0.6725 | R= -0.08  p=0.8324 | R= -0.04  p=0.8976 |
| Thalamus  small VOI | R=0.25  p=0.4876 | R= -0.34  p=0.1982 | R= 0.39  p=0.1532 | R=0.47  p=0.1619 | R=0.17  p=0.5545 |
| FC  full-size VOI | **R=0.96**  **p<0.0001** | **R= -0.83**  **p<0.0001** | **R= -0.71**  **p=0.0033** | **R= -0.65**  **p=0.0426** | **R=0.74**  **p=0.0017** |
| FC  small VOI | **R=0.96**  **p<0.0001** | **R= -0.83**  **p<0.0001** | **R= -0.69**  **p=0.0048** | R= -0.60  p=0.0632 | **R=0.68**  **p=0.0053** |

R: correlation coefficient


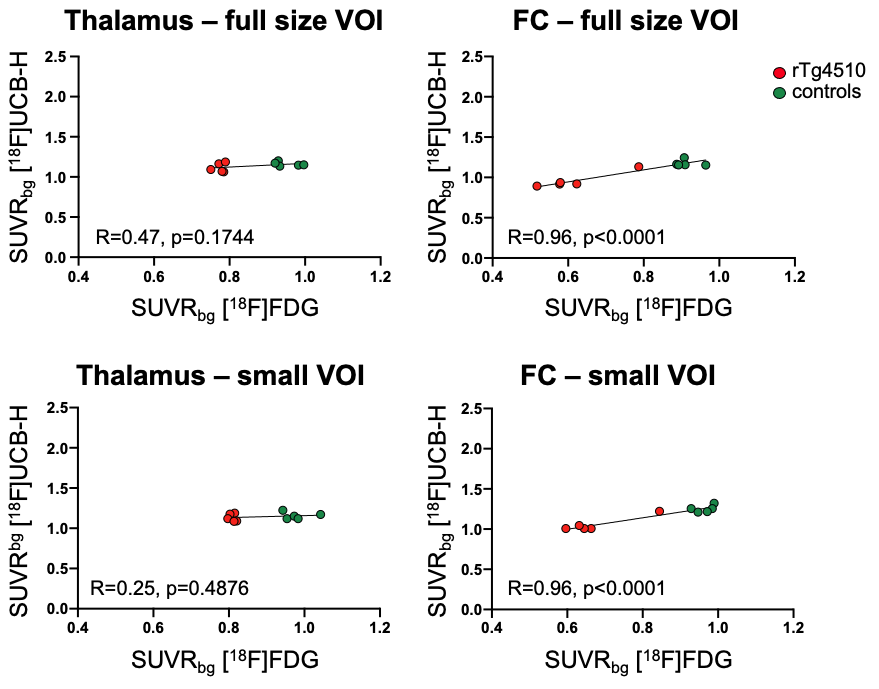


Supplemental Fig. S4: Relationship between glucose metabolism and synaptic density in the thalamus and the frontal cortex (FC). Significant correlations were found in the FC, but not in the thalamus.


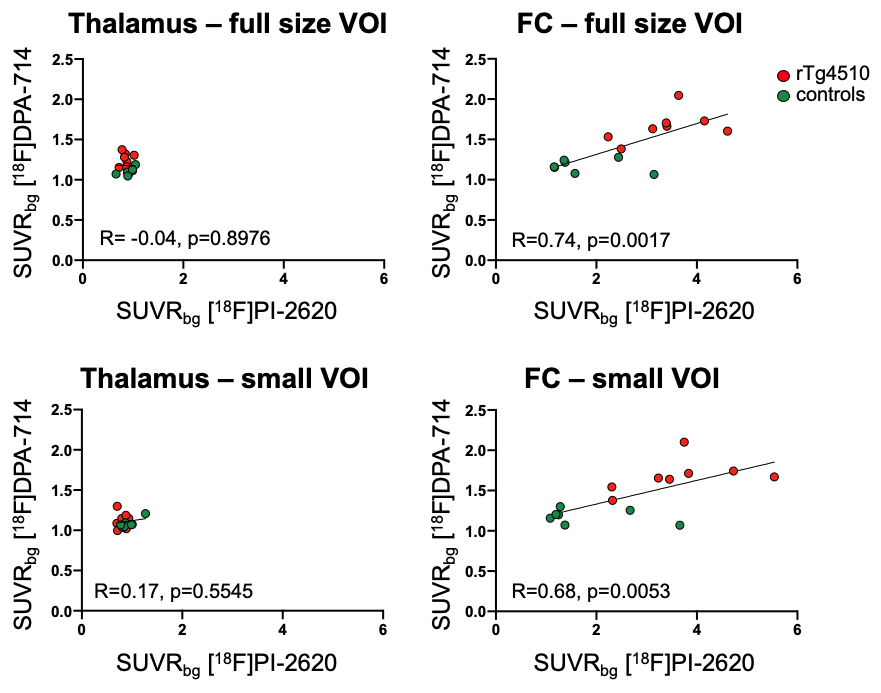


Supplemental Fig. S5: Relationship between tau deposition and neuroinflammation in the thalamus and the frontal cortex (FC). Significant correlations were found in the FC, but not in the thalamus.

2.) Voxel-wise analysis where brains of transgenic mice were increased in size.

In addition to the VOI-based approach, the voxel-wise analysis was also corrected for atrophy. To this end, the brains of rTg4510 mice were linearly enlarged by 13% in the transverse plane, and by 7% in the axial plane. The analysis shown in the main part of the manuscript in Fig. 2 was repeated with the enlarged images (Supplemental figure S6). The results are similar, except for [^18^F]PI-2620, where significant differences between rTg4510 mice and controls were less extensive. Furthermore, the voxel-wise correlation analysis shown in Fig. 3 was repeated with the enlarged images as well (Supplemental figure S7). The correlations of [^18^F]FDG were very similar, while the correlations of [^18^F]PI-2620 were less extensive after atrophy correction.

We conclude that the different approaches of atrophy correction changed details of the results, but the main findings and inferences remained the same.


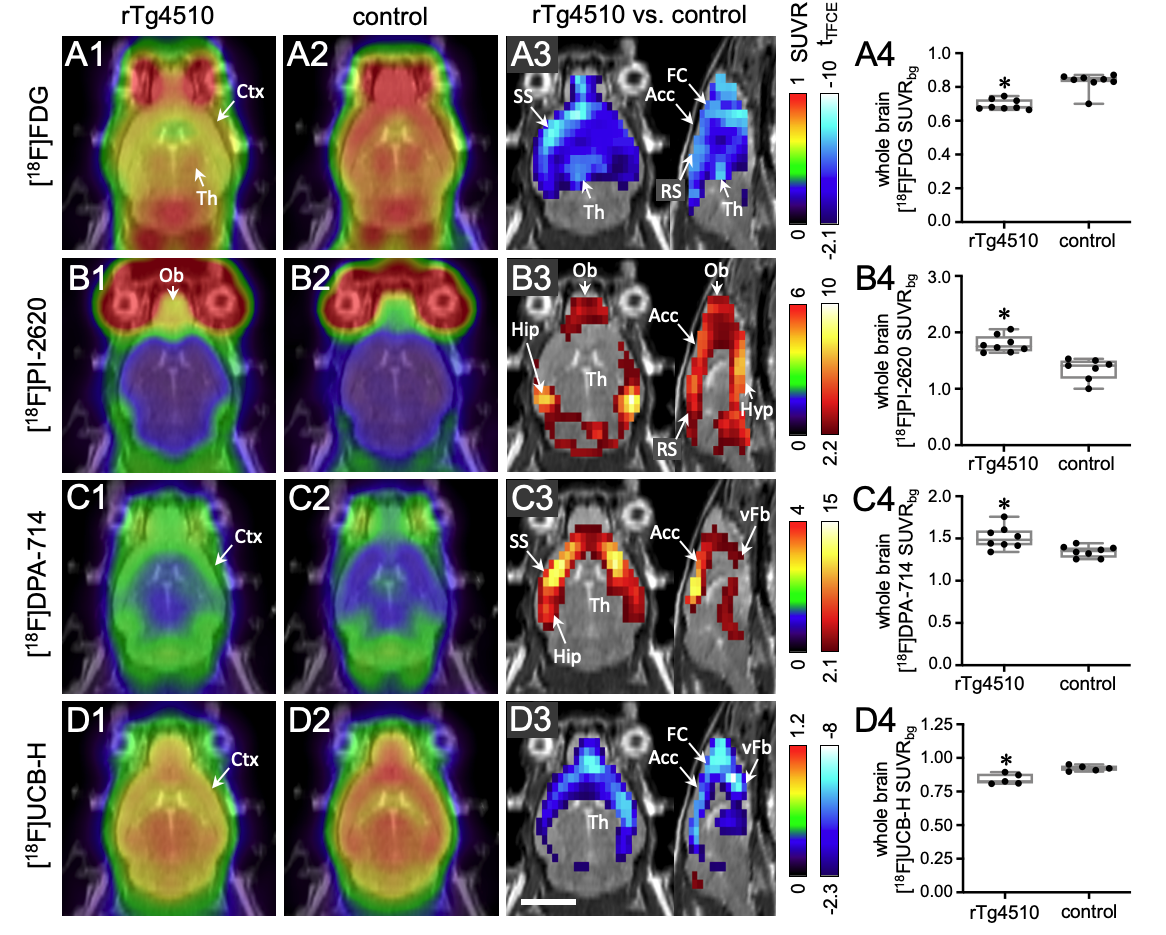


Supplemental Fig. S6: Multitracer imaging of rTg4510 mice and non-transgenic littermates (controls) after atrophy correction.

Each row A-D represents a different tracer. Column 1: Mean horizontal images of rTg4510 mice (n= 5 to 8). Column 2: Mean images of controls (n= 5 to 8). Column 3: Voxel-wise comparison between rTg4510 and control mice (t-test, corrected for multiple testing). Red and blue voxels indicate significantly (p<0.05) higher and lower tracer uptake, respectively, in rTg4510 mice after correction for multiple testing. Column 4: Comparison of whole brain tracer uptake (t-test).

*: t-test, p<0.05. Scale bar: 5 mm. Abbreviations: Acc: anterior cingulate cortex, Ctx: cortex, FC: frontal cortex, Hip: hippocampus, Hyp: hypothalamus, Ob: olfactory bulb, RS: retrosplenial cortex, SS: somatosensory cortex, Th: thalamus, vFb: ventral forebrain.


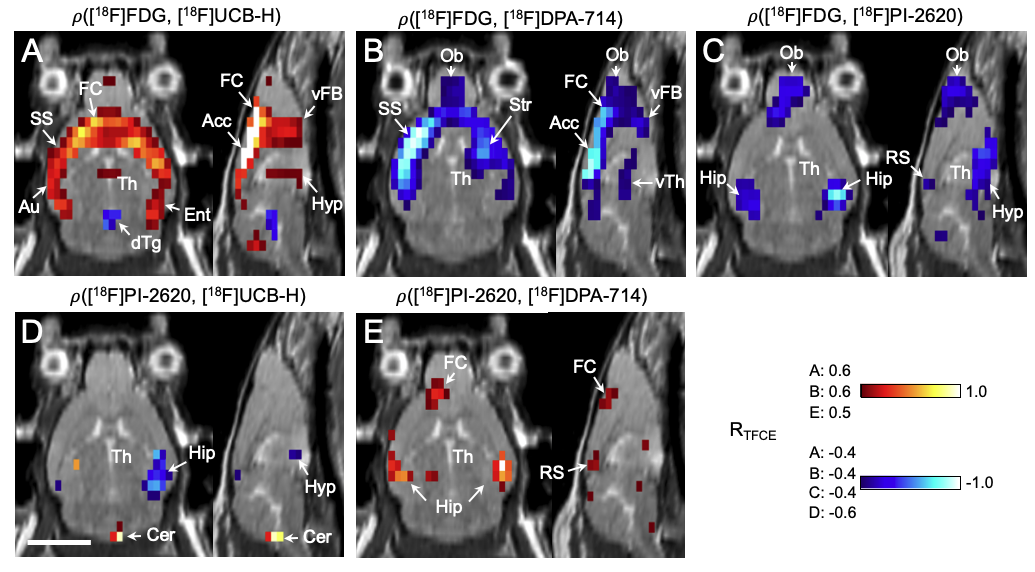


Supplemental Fig. S7: Correlation between uptake of different tracers in rTg4510 mice and controls (pooled) after atrophy correction.

Datasets of different tracers were correlated with each other using a Pearson correlation test (significance level p<0.05, corrected for multiple testing). The resulting correlation maps show for each voxel if the two respective tracers are positively (red) or negatively correlated (blue), or show no relationship at all (grey). The strength of correlation is coded by hue, with light colors reporting strong correlation (R near ±1) and dark colors indicating weak correlation (R near the significance threshold). The respective significance thresholds are noted on the left side of the color bars, and the brain areas with significant clusters are labeled. [^18^F]FDG (glucose metabolism) was correlated with (A) [^18^F]UCB-H (synaptic density), (B) [^18^F]DPA-714 (TSPO expression), and (C) [^18^F]PI-2620 (tau deposition). [^18^F]PI-2620 (tau deposition) was correlated with (D) [^18^F]UCB-H (synaptic density) and (E) [^18^F]DPA-714 (TSPO expression).

Scale bar: 5 mm. Abbreviations: Acc: anterior cingulate cortex, Au: auditory cortex, BF: basal forebrain, dTg: dorsal tegmental nucleus, dTh: dorsal thalamus, Ent: entorhinal cortex, FC: frontal cortex, Hip: hippocampus, Hyp: hypothalamus, Ob: olfactory bulb, PAG: periaqueductal grey, R_TFCE_: correlation coefficient, thresholded with a TFCE procedure, RS: retrosplenial cortex, SS: somatosensory cortex, Str: striatum, Th: thalamus, vFb: ventral forebrain, vTh: ventral thalamus.

Conclusion

Both methods used for atrophy correction (i.e. using smaller VOIs and increasing the size of rTg4510 brains) yielded results comparable with the original data. Our conclusion that two regionally distinct forms of molecular pathology are present in rTg4510 mice is therefore still supported by the atrophy-corrected data.

**Spectral characterization of DPA-714 and the DPA-714 precursor**

The corresponding precursor for radiolabeling, *N*,*N*-diethyl-2-{2-[4-(2-toluenesulfonyloxyethoxy)phenyl]-5,7-dimethylpyrazolo[1,5-a]pyrimidin-3-yl}acetamide, and DPA-714 were both produced according to the published methods (Damont et al., 2008, doi: 10.1002/jlcr.1523)

Spectral characterization of DPA-714 and the DPA-714 precursor is provided below.

*N*,*N*-Diethyl-2-{2-[4-(2-toluenesulfonyloxyethoxy)phenyl]-5,7-dimethylpyrazolo[1,5-a]pyrimidin-3-yl}acetamide (DPA-714 precursor)

^1^H NMR [400 MHz, (CD_3_)_2_CO] δ 7.88 – 7.83 (m, 4H), 7.53 – 7.43 (m, 2H), 6.97 – 6.92 (m, 2H), 6.72 (d, J = 0.9 Hz, 1H), 4.47 – 4.41 (m, 2H), 4.33 – 4.25 (m, 2H), 3.64 (q, J = 7.1 Hz, 2H), 3.39 (q, J = 7.1 Hz, 2H), 2.71 (s, 3H), 2.50 (s, 3H), 2.46 (s, 3H), 1.26 (t, J = 7.1 Hz, 3H), 1.09 (t, J = 7.1 Hz, 3H).

^13^C NMR [101 MHz, (CD_3_)_2_CO] δ 170.20, 159.30, 158.12, 155.00, 148.48, 145.96, 145.57, 134.19, 130.89, 130.58, 128.78, 128.14, 115.21, 108.85, 101.99, 69.74, 66.49, 42.82, 41.01, 28.59, 24.61, 21.55, 16.65, 14.81, 13.54.

ESI-MS: positive mode: 551.22 ([M + H]^+^)


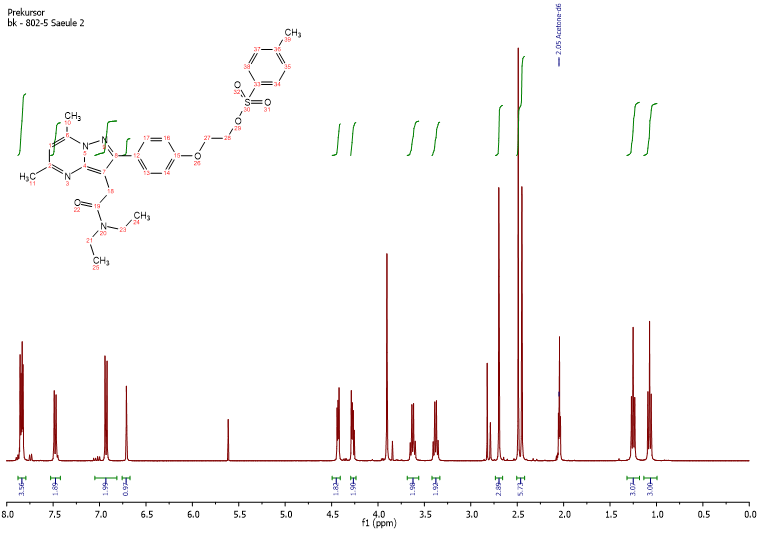


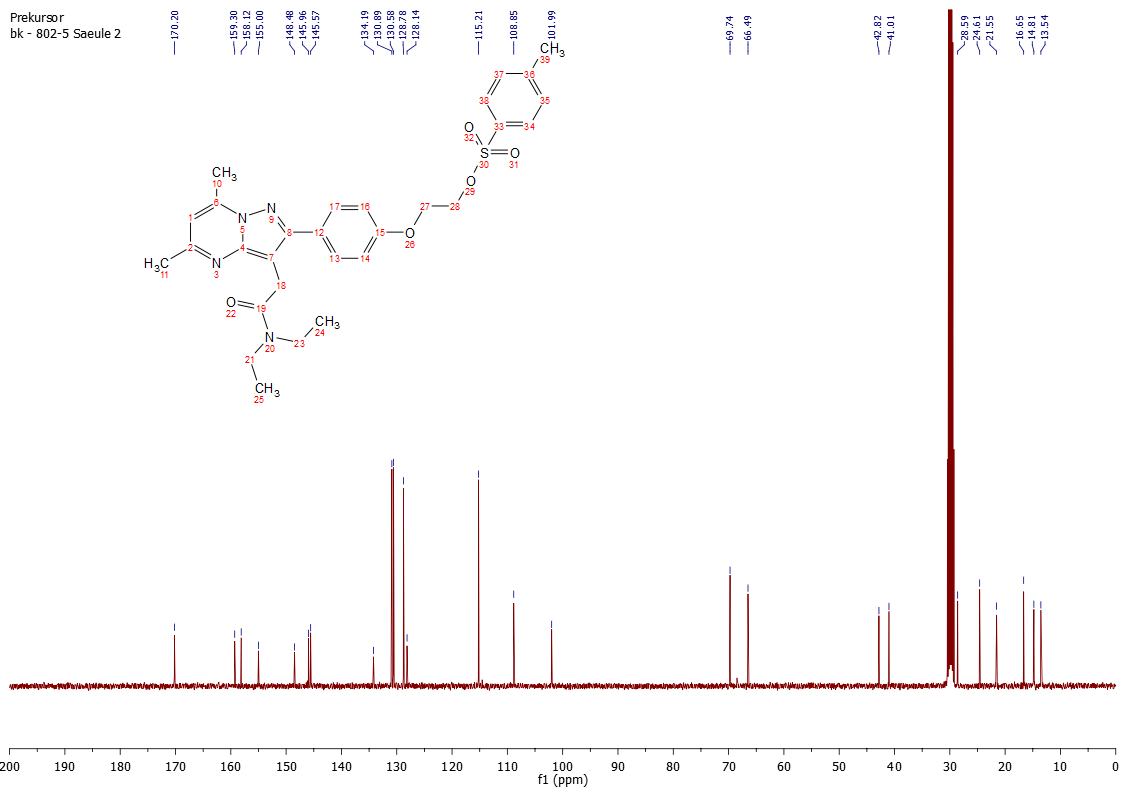

*N*,*N*-Diethyl-2-{2-[4-(2-fluoroethoxy)phenyl]-5,7-dimethylpyrazolo[1,5-a]pyrimidin-3-yl}acetamide (DPA-714 reference compound)

^1^H NMR [400 MHz, (CD_3_)_2_CO] δ 7.94 – 7.86 (m, 2H), 7.10 – 7.03 (m, 2H), 6.72 (d, 1H), 4.89 – 4.83 (m, 1H), 4.77 – 4.72 (m, 1H), 4.37 (ddd, J = 9.3, 4.6, 3.3 Hz, 1H), 4.29 (ddd, J = 12.5, 6.2, 4.9 Hz, 1H), 3.63 (q, J = 7.1 Hz, 2H), 3.39 (q, J = 7.1 Hz, 2H), 2.71 (d, J = 0.9 Hz, 3H), 2.50 (s, 3H), 1.26 (t, J = 7.1 Hz, 3H), 1.08 (t, J = 7.1 Hz, 3H).

^13^C NMR [101 MHz, (CD_3_)_2_CO] δ 170.21, 159.75, 158.11, 155.06, 148.49, 145.58, 130.66, 128.04, 115.20, 108.83, 102.00, 83.79, 82.12, 68.23 (d, J = 19.8 Hz), 41.91 (d, J = 182.4 Hz), 28.60, 24.61, 16.65, 14.79, 13.53.

^19^F NMR [1376 MHz, (CD_3_)_2_CO] δ -224.12.

ESI-MS: positive mode: 798,15 ([2M + H]^+^), 399.17 ([M + H]^+^).


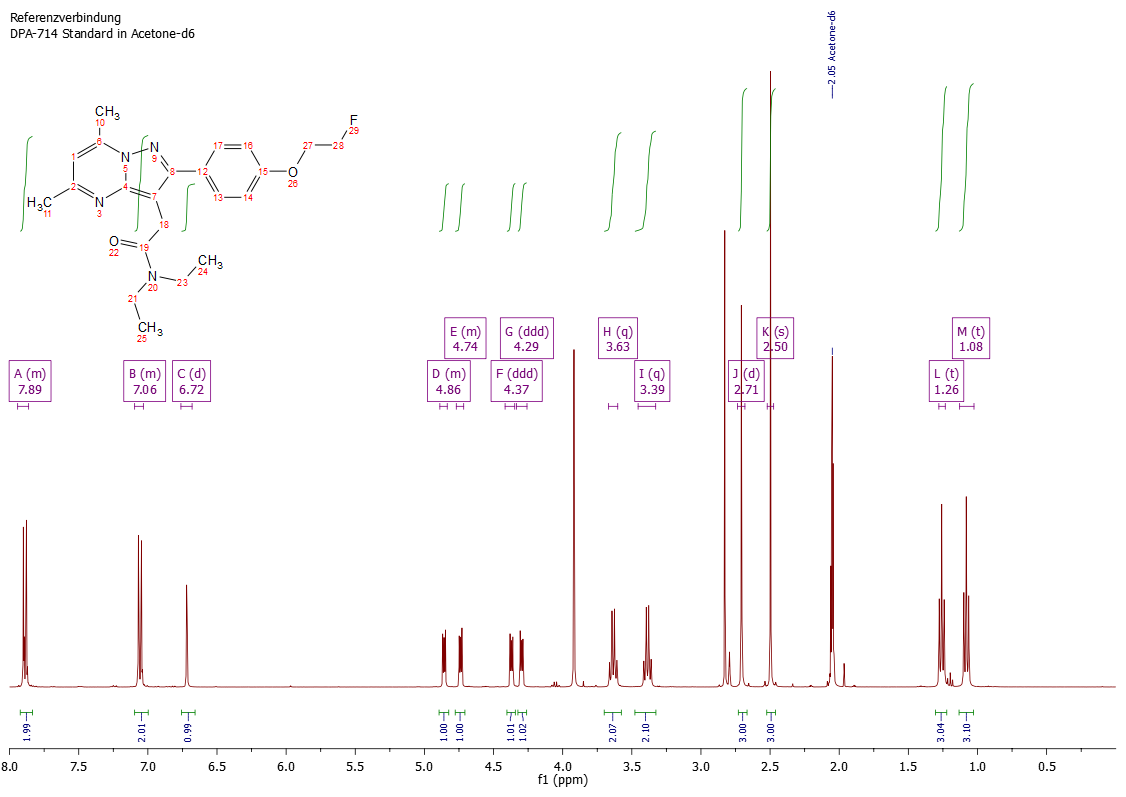


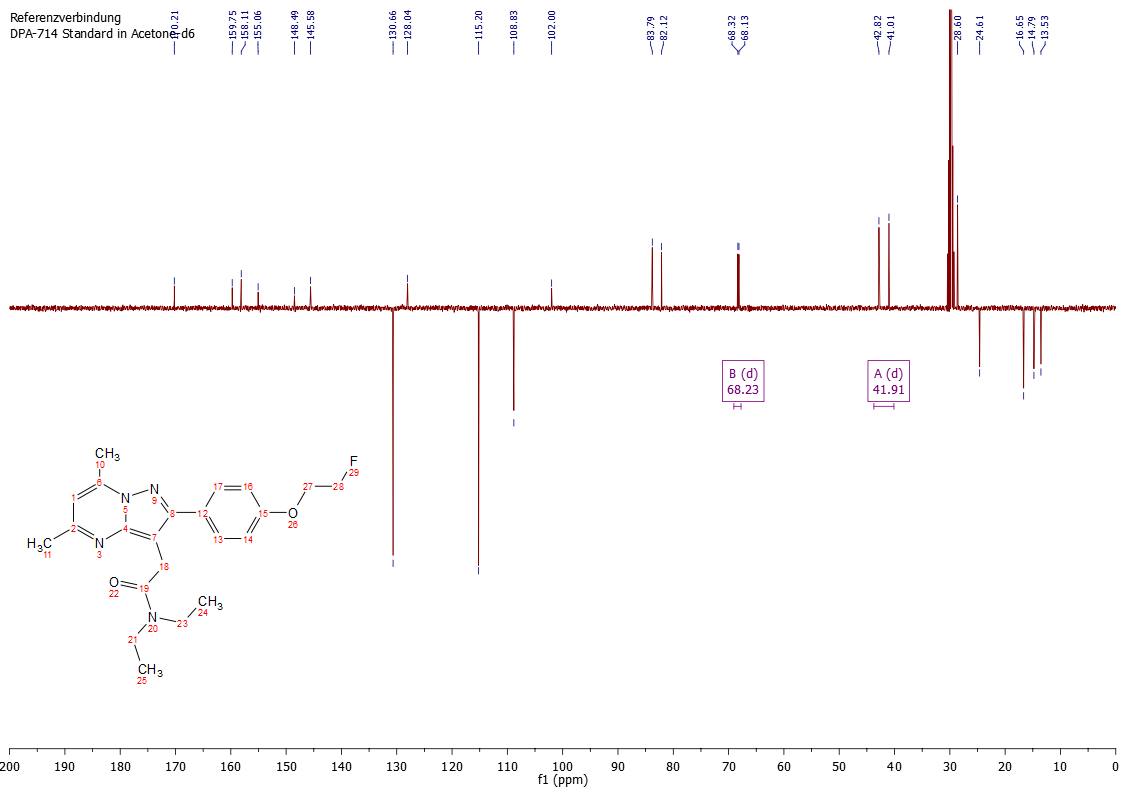


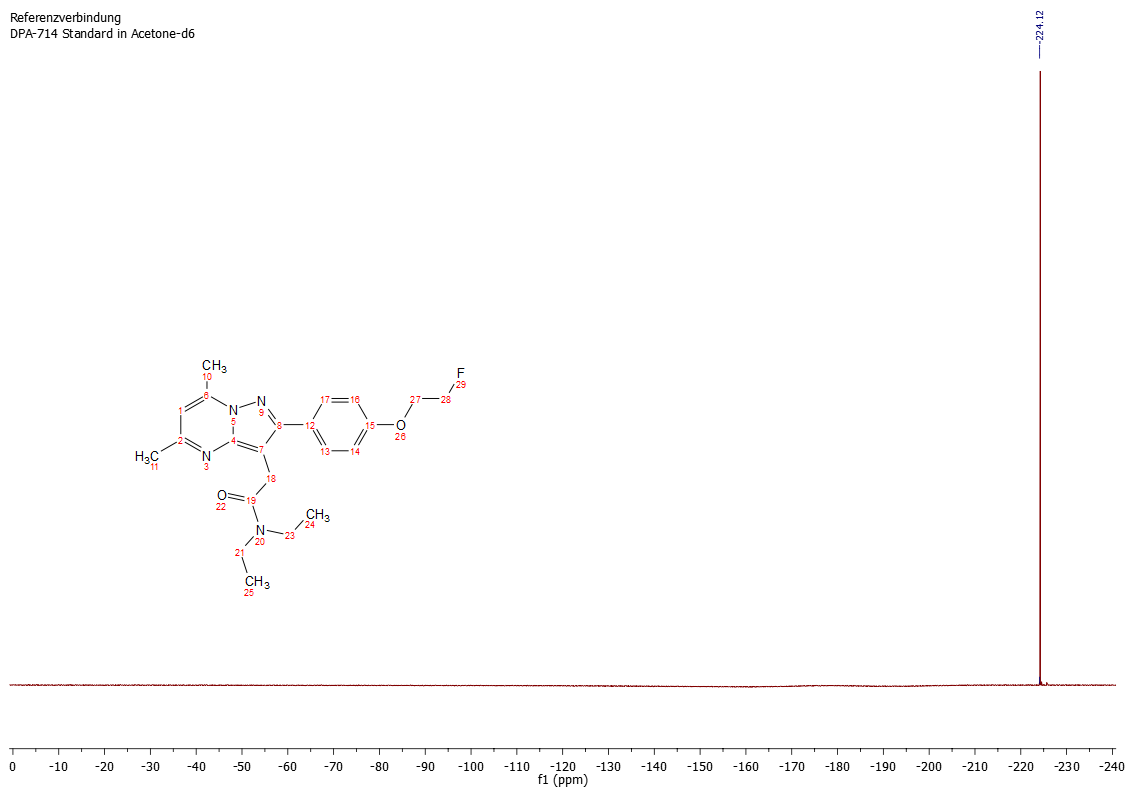

Supplement: Supplementary file 1 — Supplementary file1 (DOCX 2.73 MB) [file 12035_2022_2793_MOESM1_ESM.docx]
